# Electrosprayed Ultra-Thin Coating of Ethyl Cellulose on Drug Nanoparticles for Improved Sustained Release

**DOI:** 10.3390/nano10091758

**Published:** 2020-09-06

**Authors:** Wei-Dong Huang, Xizi Xu, Han-Lin Wang, Jie-Xun Huang, Xiao-Hua Zuo, Xiao-Ju Lu, Xian-Li Liu, Deng-Guang Yu

**Affiliations:** 1 School of Chemistry and Chemical Engineering, Hubei Polytechnic University, Huangshi 435003, China; neweydong@hbpu.edu.cn (W.-D.H.); 211005@hbpu.edu.cn (X.-H.Z.); 2 Hubei Key Laboratory of Mine Environmental Pollution Control and Remediation, School of Environmental Science and Engineering, Hubei Polytechnic University, Huangshi 435003, China; 211041@hbpu.edu.cn (H.-L.W.); huangjiexun@hbpu.edu.cn (J.-X.H.); 3 School of Materials Science and Engineering, University of Shanghai for Science and Technology, Shanghai 200093, China; 192432592@st.usst.edu.cn

**Keywords:** coaxial electrospraying, surface design, coating process, sustained release, release kinetic, nanoparticles

## Abstract

In nanopharmaceutics, polymeric coating is a popular strategy for modifying the drug release kinetics and, thus, new methods for implementing the nanocoating processes are highly desired. In the present study, a modified coaxial electrospraying process was developed to formulate an ultra-thin layer of ethyl cellulose (EC) on a medicated composite core consisting of tamoxifen citrate (TAM) and EC. A traditional single-fluid blending electrospraying and its monolithic EC-TAM nanoparticles (NPs) were exploited to compare. The modified coaxial processes were demonstrated to be more continuous and robust. The created NPs with EC coating had a higher quality than the monolithic ones in terms of the shape, surface smoothness, and the uniform size distribution, as verified by the SEM and TEM results. XRD patterns suggested that TAM presented in all the NPs in an amorphous state thanks to the fine compatibility between EC and TAM, as indicated by the attenuated total reflection (ATR)-FTIR spectra. In vitro dissolution tests demonstrated that the NPs with EC coating required a time period of 7.58 h, 12.79 h, and 28.74 h for an accumulative release of 30%, 50%, and 90% of the loaded drug, respectively. The protocols reported here open a new way for developing novel medicated nanoparticles with functional coating.

## 1. Introduction

During the past several decades, polymeric excipients have provided numerous strategies for developing new dosage forms with all sorts of drug-controlled release profiles [1,2,3,4]. Among them, polymeric coating is one of the most popular strategies for modifying the drug release kinetics in both the traditional pharmaceutical industry and also the advanced laboratory nanomethods [5,6,7,8,9,10,11,12,13]. Particularly in nanopharmaceutics, the simple and robust nanocoating methods are highly desired [14,15,16,17,18]. In general, there are two kinds of nanofabrication routes, i.e., bottom-up and top-down [19,20,21]. The bottom-up routes (such as molecular self-assembly) have their advantages in production, such as accurate manipulation and adjusting of the products’ sizes. However, they often comprise time-consuming and multiple-step processes and, thus, often have the difficulties of production on a large scale [22]. In comparison, the top-down routes (such as electrospinning and electrospraying) hold the fine promises for creating nanoproducts in a single-step manner and on a large scale [23,24,25,26,27,28,29,30,31,32] and, thus, they should provide new strategies for implementing nanocoating on solid medicated nanoparticles [33,34,35,36,37].

In a broad meaning, the coating of polymer on a medicated core represents a shell section of a core-shell structure [38,39,40,41,42,43]. Electrospinning, as a brother technique of electrospraying, has broadly demonstrated its great power in creating core-shell nanofibers [44,45]. In literature, coaxial electrospinning [44,45,46], modified coaxial electrospinning [46], modified triaxial electrospinning [47,48,49,50], and other complicated processes [51] are reported to generate a thin layer of coating one the core medicated nanofibers. The created core-shell nanostructures with a polymer nanocoating are verified to be very useful for manipulating the drug-loading amount and also their sustained release profiles. Enlightened by electrospinning, electrospraying (particularly the coaxial electrospraying) should play its important role in creating nanocoating on the solid medicated nanoparticles.

For a successful coaxial electrospraying, the traditional concept is that the shell working fluid must be solidifiable (meaning that solid particles can be produced after being subjected to a single-fluid electrospraying process) to form a solid core-shell nanostructure; the core fluid either has or does not have solidifiable properties. This knowledge was achieved from the first study about traditional coaxial electrospraying [52]. However, Liu et al. successfully demonstrated a modified coaxial electrospraying process, in which the shell fluid was unsolidifiable organic solvent. The shell solvent changed the interfaces between the atmosphere and the polymeric working fluid through inserting a middle solvent layer during the spraying process. The resultant particles were verified to have higher quality in terms of particles sizes and size distribution, few satellites, and smooth surface [53]. Later, this modified coaxial process was successfully explored to round up the polymeric nanoparticles for better sustained release [34].

Going a further step along the previous development of modified coaxial electrospraying, the present study developed a new, modified coaxial electrospraying, in which a dilute ethylcellulose (EC) solution without solidifiable property was exploited as the shell working fluid to co-spray with a solidifiable core fluid containing a higher concentration of EC and drug. An anti-cancer drug, tamoxifen citrate (TAM), was utilized as a model drug to study the drug release kinetics. The morphology of the created particles, the thicknesses of the nanocoatings, and their performances in manipulating the drug sustained release kinetics were investigated in detail.

## 2. Materials and Methods

### 2.1. Materials

TAM (purity of 98%, white crystalline powders) was bought from Hua-Shi Pharmacy (Shanghai, China). EC was obtained from Shandong Fine Chemical Co., Ltd. (Jinan, China). Dichloromethane (DCM) and anhydrous ethanol were purchased from Shanghai Chemical Analysis Factory (Shanghai, China). All other components are analytical reagents, and water was double-distilled before use. 

### 2.2. Modified Coaxial Electrospraying

A solution having solidifiable property was prepared by dissolving 10 g EC and 3 g TAM in a mixture of ethanol and DCM (1:1, v/v), meaning a concentration of 10% (w/v) EC and 3% (w/v) for them, respectively. This solution was exploited as the core working fluid. An unsolidifiable solution consisting of 2% (w/v) EC in a mixture of ethanol and DCM (1:1, v/v) was used as the shell fluid. Two sorts of nanoparticles, referred to as E1 and E2, were prepared at an applied voltage between 16–18 kV. For all preparations, the particle-collected distance was fixed at 15 cm. The electrospraying processes were recorded using a digital camera (PowerShot A640, Tokyo, Japan). Other parameters are given in Table 1. 

### 2.3. Morphology of the Prepared Nanoparticles

Morphology and diameter of the fabricated NPs were studied and evaluated using a scanning electron microscope (SEM, Quanter 450, FEI, Hillsboro, OR, USA). The samples were sputtered with a thin layer of gold. Then the images were visualized and taken under an applied voltage of 20 kV. The average diameters of the NPs were determined by ImageJ software (National Institutes of Health, Bethesda, MD, USA) by randomly selecting about 50 particles. A transmission electron microscope (TEM, JEM 2100F, JEOL, Tokyo, Japan) was used to evaluate the inner structure of the prepared NPs.

### 2.4. Physical State of the Components and Their Compatibility

X-ray diffraction (XRD) patterns were recorded over the range 2*θ* from 5° to 60° using a Bruker X-ray diffractometer (Karlsruhu, Germany) with CuKα radiation. The X-rays were emitted at 40 kV and 30 mA. The presence of components, physical/chemical interactions, and drug–excipient compatibility were evaluated by using a Spectrum 100 FTIR Spectrometer (PerkinElmer, Waltham, MA, USA) at a range of 500–4000 cm^−1^ at a resolution of 2 cm^−1^.

### 2.5. In Vitro Dissolution Tests

The drug release profiles from the NPs were assessed using a paddle method according to the Chinese Pharmacopoeia (2015 Edition). A weight of 100 mg NPs was placed into 900 mL physiological saline (pH = 7.0). The dissolution media were kept at 37 °C and a rotation rate of 50 rpm. At predetermined time points, a 5.0-mL aliquot was withdrawn and 5.0 mL of fresh physiological saline was added. The amounts of TAM released were measured at *λ*_max_ = 272 nm using a UV-VIS spectrophotometer (UV-2102PC, Unico Instrument Co. Ltd., Shanghai, China). The experimental results were reported as mean ± standard deviation. All experiments were repeated six times. 

## 3. Results and Discussion

### 3.1. Implementation of the Modified Coaxial Electrospraying

**Figure 1:**
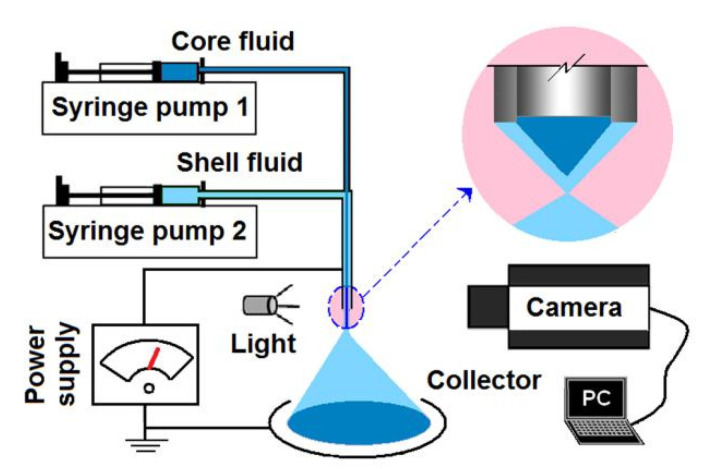
A schematic showing the main components of a modified coaxial system.

A schematic showing the main components of a modified coaxial system is given in Figure 1. Just as a coaxial electrospraying system or even a traditional single-fluid electrospraying system [54,55,56], the modified coaxial system, as an electrohydrodynamic atomization (EHDA) place, has the prerequisite four parts: A power supply for “electro-”, two syringe pumps for “hydro-”, a spraying head for the beginning of “dynamic” process, and a collector for collecting the resultant products of “atomization”. Other auxiliary parts (such as lights/camera/personal computer for observing the working processes, hot air blower for helping solidification) can be added to the system when they are necessary for a successful preparation of electrosprayed solid particles [57,58].

**Figure 2:**
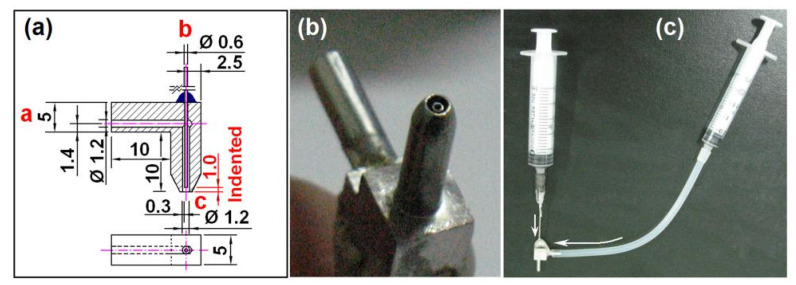
The homemade concentric spraying head for carrying out the modified coaxial processes: (**a**) Design details of the concentric spinneret with an indented section for fine encapsulation; (**b**) a digital picture about the concentric spraying head; (**c**) the connection of spraying head with two syringes.

Among the four necessary parts, the spraying head is the most important section because it is a convergent point of electrical energy and fluid (or fluids), and it has the important role of guiding the working fluid (or fluids) into the electrical field for implementing an electrospraying process [59,60,61]. Thus, it is common sense that the working processes are often termed by the structures of the spraying heads. For example, a coaxial electrospraying is named due to a concentric spraying head that is utilized to organize the two working fluids. A side-by-side electrospraying (or electrospinning) is called because a side-by-side spraying head (or spinneret) is exploited to lead the double fluids into the electrical field in a side-by-side manner [62,63,64]. Shown in Figure 2 are the contents about the design (a), digital image (b), and connection with two syringes that can be utilized to hold the shell and core working fluids (c). The slight indenting of the core capillary about 0.1 mm should be favorable for a fine encapsulation of the shell fluid on the core fluid. 

**Figure 3:**
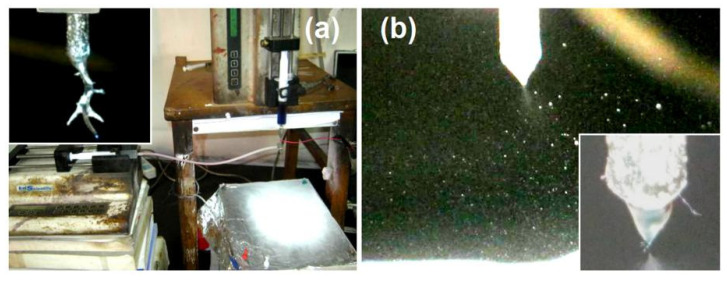
Observations about the modified coaxial electrospraying: (**a**) A digital picture about the whole apparatus and the preparation of monolithic nanoparticles E1, the upper-left inset showing a clogging phenomenon when the core EC-TAM blended fluid was subjected to electrospraying alone. (**b**) A digital image exhibiting the preparation process of nanoparticles E2 having an EC coating, the lower-right inset showing a typical compound Taylor cone.

The fabrication processes of the monolithic nanoparticles E1 and nanoparticles E2 with an EC coating are shown in Figure 3. When the shell working fluid of 2% (w/v) EC was turned off, the working process was essentially a single-fluid blending electrospraying. The core blended solutions had a nice, solidifiable property under the treatment of electrospraying. The process is shown in Figure 3a. Although 1 × 10^−3^ mg/mL methylene blue was added into the core fluid and the working fluid showed a blue color, the collected particles still had almost a white color. During the preparation process, the spraying head needed to be manually cleaned now and then because of the clogging from the semisolid substances. A typical image of them is shown in the upper-left inset image of Figure 3a. It should be attributed to the solvent mixture of ethanol and DCM, which are very easy to be evaporated and also the relatively high concentration of EC.

However, the fabrication processes of nanoparticles E2 were robust and continuous. Figure 3b shows a typical working process of the electrospraying. The classic three steps can be viewed clearly, i.e., the formation of a compound Taylor cone of the shell and core working fluids, a convergent point or a straight fluid jet, and a Coulomb explosion region showing the repelling and division of the charged liquids until they were solidified to reach the collector. A typical compound Taylor cone can be recorded under a magnification of 12 with an opposite light source and the additive methylene blue as a color marker, which is given in the lower-right inset of Figure 3b.

### 3.2. The Morphology and Nanocoating Structure Studies

**Figure 4:**
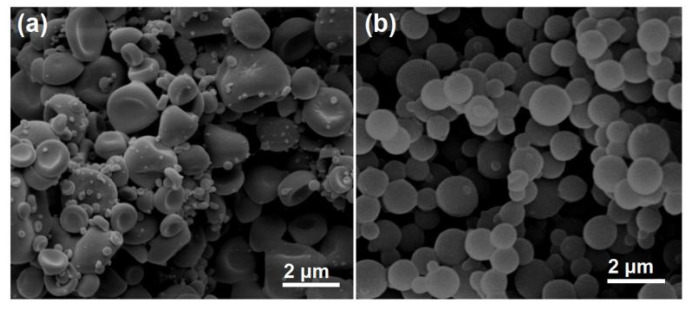
The SEM images of the prepared monolithic NPs using a single-fluid blended electrospraying (**a**) and NPs with EC coating using the modified coaxial electrospraying (**b**).

Just as anticipated, the monolithic nanoparticles E1 prepared using the single-fluid blended electrospraying showed a flat image with a center indented region, which is exhibited in Figure 4a. Meanwhile, there were many satellites clinging on the electrosprayed particles. The estimated diameters of over 50 particles had an average value of 1.34 ± 0.27 μm. Apparently, these particles had a poor quality in terms of the shape, surface smoothness, and the uniform size distribution. The manual intervention on the preparation processes, the clogging phenomena, and also the unstable evaporation of DCM and ethanol co-acted to create these “ugly” nanoproducts. In sharp contrast, the prepared nanoparticles E2 with EC coating show a high quality in Figure 4b. These particles had a round morphology, a smooth surface, and also a relatively uniform size distribution of 0.87 ± 0.14 μm. Meanwhile, the numbers of satellites were very small.

**Figure 5:**
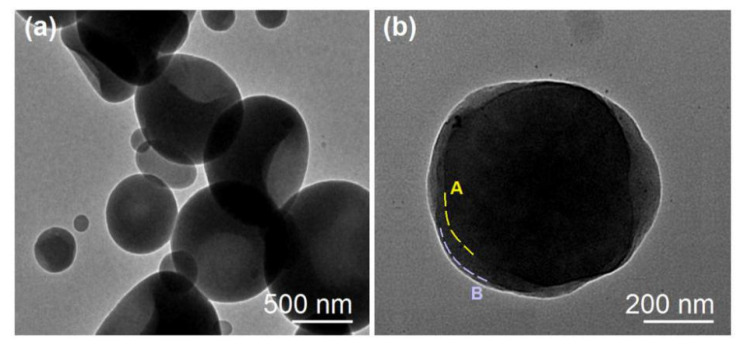
The TEM images of the of the prepared monolithic NPs using a single-fluid blended electrospraying (**a**) and NPs with EC coating using the modified coaxial electrospraying (**b**).

To evaluate the inner structures of those electrosprayed particles, TEM images were achieved and are shown in Figure 5. The monolithic NPs E1 prepared from the single-fluid blended electrospraying had an irregular shape with uneven gray levels (Figure 5a). The gray levels of TEM images under a bright field resulted from three sections, i.e., the element, the density, and the thickness. Apparently, the indented sections in the particles often had a small thickness and, thus, correspondingly had a lighter gray level. In contrast, nanoparticles E2 from the modified coaxial electrospraying processes had an obvious core-shell nanostructure (Figure 5b). The core sections had a heavier gray level than the shell section, which should be attributed to the different thickness of them and also their densities. The drug molecules can be distributed in the voids of the entanglements among the EC molecules and, thus, they can elevate the density of core sections. The thickness of the shell sections was estimated about 10–70 nm. In some places, there were transition regions between the core and shell sections, as indicated by dash lines A and B in Figure 5b. This phenomenon suggests that some drug molecules might diffuse from the core fluids to the shell fluids during the electrospraying processes.

### 3.3. The Physical State of the Drug and the Compatibility of the Components

**Figure 6:**
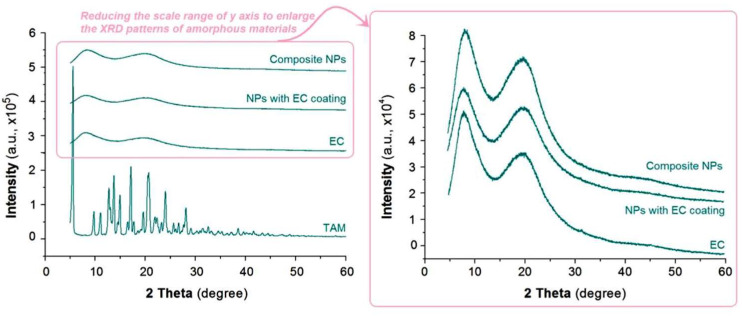
The XRD patterns of the crude materials and also their nanoparticles.

Shown in Figure 6 are XRD patterns of the crude materials (TAM and EC) and their nanoparticles E1 and E2. The patterns of TAM suggest that the raw TAM particles were typical crystalline due to the many sharp peaks responding to Bragg diffraction [49]. In contrast, the polymer EC had no responding peaks except two halos, suggesting that it was an amorphous material [47]. The nanoparticles from the two different electrospraying processes had a similar XRD pattern, just as the curve of EC. These phenomena indicate that the nanoparticles were amorphous nanocomposites. In other words, the drug TAM molecules co-existed with EC molecules in a molecularly dispersive state. The EC molecules acted as the space blocking agents to prevent the contact of TAM molecules and re-crystallization. The molecular dispersion state of EC and TAM molecules was a result of the extremely fast drying process of the electrospraying, by which the highly uniform state of TAM and EC in the working fluid was propagated into the solid nanoparticles, regardless of their morphologies and inner structures. Similar results are frequently reported about electrospun medicated nanofibers [25,38].

**Figure 7:**
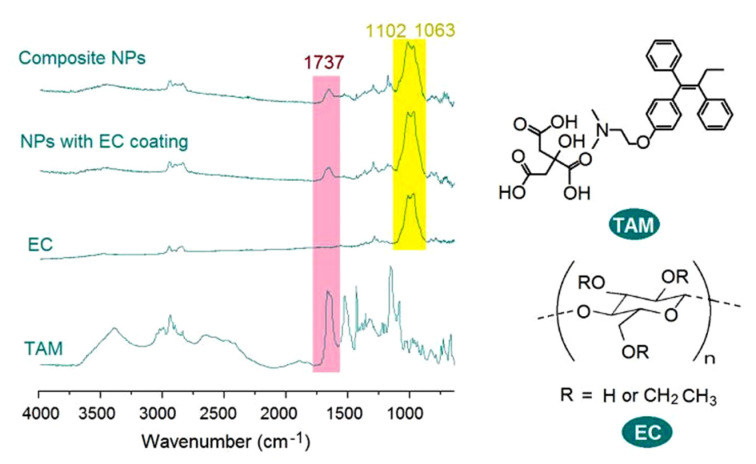
The attenuated total reflection (ATR)-FTIR spectra of the crude materials (EC and TAM) and also their nanoparticles and the molecular formula of TAM and EC.

Attenuated total reflection ATR-FTIR spectra of the raw materials EC and TAM and also their nanoparticles are exhibited in Figure 7. The most important characteristic peak of TAM was at 1737 cm^−1^, which was a response from the –C=O groups. As for the EC, the strongest characteristic responses were at 1102 and 1063 cm^−1^. In their electrosprayed composite, NPs E1 and NPs E2 with EC coating, many peaks of the TAM were significantly decreased or even disappeared, which suggested that EC and TAM had good compatibility. This can be expected from their molecular formula. In a TAM molecule, there are three –C=O groups and three benzene rings. The –C=O groups can easily form hydrogen bonds with the –OH groups in the EC molecules. Meanwhile, the benzene rings can interact with long carbon chains of EC molecules through the hydrophobic interactions. Thus, these second interactions should do favor to the fine compatibility of EC and TAM and also increase the physical stability of the prepared nanoparticles.

### 3.4. The Functional Performances of the Nanocoating on the Drug Sustained Release Kinetics

**Figure 8:**
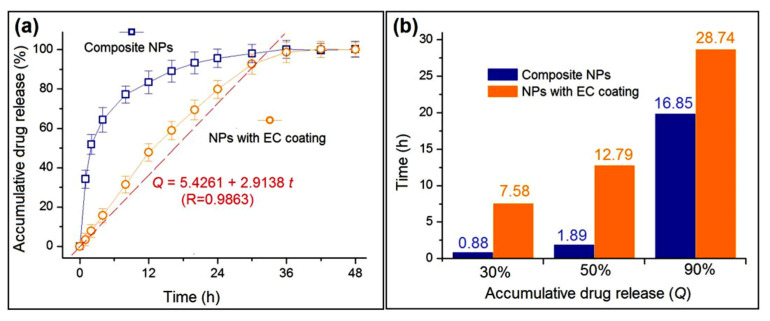
The in vitro drug sustained release performances: (**a**) Accumulative drug release as a function of drug release time and (**b**) the needed time for reaching a certain percentage of drug loaded in the created nanoparticles.

The drug sustained release profiles of the two types of NPs are included in Figure 8. Figure 8a,b shows the accumulative drug release (%) as a function of drug release time and the time needed for finishing a certain percentage of drug loaded in the created nanoparticles, respectively. The performances can be compared mainly from two aspects: The initial burst release amount and the subsequent sustained release time period. Composite NPs E1 provided a 34.2 ± 4.5% release at the first hour, a typical initial burst release because of a value over 30% [48]. In comparison, NPs with EC coating released only 3.4 ± 2.1% (Figure 8a). From another angle, a content of 30% release took 0.88 h and 7.58 h for NPs E1 and E2, respectively (Figure 8b). Thus, the NPs with EC coating completely eliminated the initial burst release due to the blank shell EC layer coating. At the sampling time point of 20 h, NPs E1 released 93.3 ± 5.5% of their cargoes, whereas NPs E2 released 92.7 ± 5.1% of the loaded drug at the time point of 30 h. The NPs with EC coating were able to provide a longer time period of drug sustained release. This can be also reflected from the time needed for finishing a release of 50% and 90%. NPs E1 needed only 1.89 h and 16.85 h, respectively, whereas NPs with EC coating took 12.79 h and 28.74 h, respectively. Additionally, the release data of NPs E2 can be regressed using the linear equation, i.e., *y* = a*x* + b. Shown in Figure 8a, the drug release percentage (*Q*) had a fine, linear relationship with the drug release time (*t*) with a high correlation coefficient R = 0.9863. The equation is *Q* = 5.4261 + 2.9138*t*. The linear release is very useful for accurately predicting the drug release profiles and, in turn, for the personal therapeutics in the future. 

**Figure 9:**
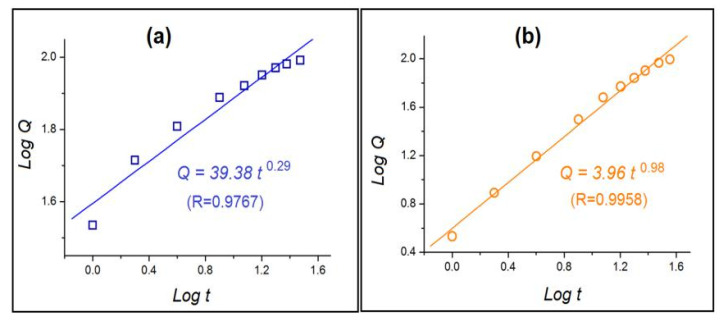
The regressed equations of the drug release data of nanoparticles E1 and E2 based on the Peppas equation: (**a**) Composite NPs E1, (**b**) NPs with EC coating E2.

To disclose the influences of EC coating on the drug release kinetics, the release data of NPs E1 and E2 were treated using Peppas equation [65,66], i.e., *Q* = k*t*^n^, where *Q* is the drug release amount, *t* is the release time, k is a constant, and n is an indicating constant about the drug release mechanism. The common knowledge is that n ≤ 0.45 suggests a Fickian diffusion mechanism, n ≥ 0.9 suggests an erosion mechanism, and 0.45 ≤ n ≤ 0.90 suggests a combined mechanism. The regressed equations for NPs E1 (Figure 9a) and E2 (Figure 9b) are *Q* = 39.38*t*^0.29^ (R = 0.9767) and *Q* = 3.96*t*^0.98^ (R = 0.9958), respectively. Thus, NPs E1 released the drug through a typical Fickian diffusion mechanism, suggested by an n value of 0.29 < 0.45. This can be expected because EC is an insoluble drug and the drug molecules must gradually penetrate and diffuse through the EC matrices to reach the bulk dissolution media. However, the drug release mechanism for the NPs E2 with EC coating was suggested to be an erosion mechanism, owing to a value of n = 0.98 > 0.90. This is not true. The reason should be that the prerequisite condition of Peppas equation is that the drug molecules must be uniformly distributed all over the matrices. Thus, the blank EC shell-layer coating in NPs E2 makes the Peppas equation invalid here. In other word, Peppas equation is useful for predicting drug release mechanism from a certain matrix but is invalid for predicting drug release mechanism from the complex nanostructures. This case was also demonstrated by the core-shell, fiber-based drug depots [47]. 

### 3.5. The Mechanism of EC Coating in Adjusting the Drug Release Kinetics and the Related Perspectives

**Figure 10:**
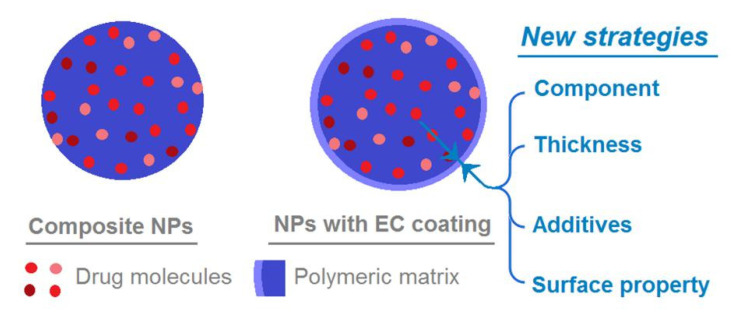
A diagram showing the functions of the ultra-thin EC coating in manipulating the drug release behaviors, and the new strategies that can be developed from the present protocols for the further investigations.

Figure 10 is a diagram explaining the functions of the ultra-thin EC coating in manipulating the drug release behaviors. For the composite NPs E1 to form the single-fluid blending electrospraying, the drug molecules are uniformly distributed within the EC matrix. Although it is soluble in a variety of organic solvents and is convenient for treating, EC is insoluble in water. This unique property has made EC become a popular coating agent for achieving oral, sustained release pharmaceutical formulations, such as tablets and pellets [67,68,69]. Thus, in the present electrosprayed NPs, the uniform distribution in NPs E1 inevitably resulted in a typical diffusion process for the drug release.

When an EC blank coating is applied on the surface of the composite NPs, it can adjust the drug release performances from the following three aspects. Firstly, the blank coating removes the presence of drug molecules on the NPs’ surface, which is very useful for eliminating the initial burst release. Secondly, the blank coating provides an additional barrier for the inner drug molecules to diffuse into the bulk solution, which is useful for prolonging the drug release time period. Thirdly, the blank EC surface should have a high hydrophobic property, which would increase the diffusion resistances of both the water molecules penetrating into the NPs and the drug molecules diffusing out from the particles to the bulk solution. Thus, the EC layer coating can modify the drug release behaviors from the NPs profoundly.

In this nano-era, NPs from all kinds of raw materials have been tried to provide the designed drug-controlled release profiles, such as polymer, lipid, inorganic, and magnetic substances [11,70,71,72,73,74]. Based on the present protocols, there are some new strategies that can be applied for developing novel polymer coating NPs. For example, a wide variety of raw materials can be explored to modify the traditional NPs through electrospraying [75,76,77]. In this study, the coating material was EC but, apparently, many other polymers and lipids can be similarly utilized to coat the medicated NPs. Meanwhile, the thicknesses of shell blank layer can be exploited as a key parameter to adjust the drug release profiles, which in turn can be manipulated by the flow rate of the shell working fluid and also its polymeric concentrations. Additives such as the pore-forming agents may be also added into the surface-coating layer, and the shell layer’s surface property may be also manipulated about its hydrophobic and hydrophilic properties for a further fine tuning of drug release profile. Particularly, some popular inorganic NPs may be improved about their biocompatibility when they are coated with polymers or lipids [78,79].

## 4. Conclusions

A modified, coaxial electrospraying was successfully implemented to form an ultra-thin EC shell layer on the medicated EC-TAM core. The diluted shell working fluid containing 2% (w/v) EC made the modified coaxial processes more continuous and robust. Meanwhile, the generated NPs with EC coating had a higher quality than the monolithic ones from the single-fluid blended process in terms of their shapes, surface smoothness, smaller size, and the uniform size distribution. XRD patterns suggested that the drug TAM presented in all the NPs in an amorphous state, owing to the good compatibility between TAM and EC. In vitro dissolution tests showed that the NPs with EC coating took time periods of 7.58 h, 12.79 h, and 28.74 h for an accumulative release of 30%, 50%, and 90% of the loaded drug, respectively. These data demonstrated that the NPs with EC coating were able to offer a better drug sustained release performance than the monolithic composite NPs, whose corresponding time periods were 0.88 h, 1.89 h, and 16.85 h. The drug molecules released formed the composite NPs through a typical Fickian diffusion mechanism, whereas the drug released from the NPs with EC coating did not obey the Peppas equation. The EC coating changed the drug molecule diffusion behaviors profoundly, which will be further investigated.

## Figures and Tables

**Table 1:** Parameters about the electrosprayed nanoparticles.

No	Process	Voltage (kV)	Flow Rate (mL/h)		Morphology/Structure ^a^	Drug Loading in the NPs (w/w%) ^b^
			Core Fluid	Shell Fluid		
E1	Single-fluid	16	1.0		Irregular/Monolithic	23.1%
E2	Modified coaxial	18	1.0	0.3	Round/Core-shell	22.1%

^a^ The morphology and structure are experimental results. ^b^ The drug loading in the nanoparticles (NPs) (w/w%) is a theoretical value calculated according to the electrospraying conditions. Because electrospraying is essentially a physical drying process and tamoxifen citrate (TAM) has a boiling point of 140–144 °C without sublimation property, the drug encapsulation efficiency can be regarded as 100% [27,46].
