# Correction: Huang et al. Electrosprayed Ultra-Thin Coating of Ethyl Cellulose on Drug Nanoparticles for Improved Sustained Release. *Nanomaterials* 2020, *10*, 1758

**DOI:** 10.3390/nano15231825

**Published:** 2025-12-02

**Authors:** Wei-Dong Huang, Xizi Xu, Han-Lin Wang, Jie-Xun Huang, Xiao-Hua Zuo, Xiao-Ju Lu, Xian-Li Liu, Deng-Guang Yu

**Affiliations:** 1 School of Chemistry and Chemical Engineering, Hubei Polytechnic University, Huangshi 435003, China; neweydong@hbpu.edu.cn (W.-D.H.); 211005@hbpu.edu.cn (X.-H.Z.); 2 Hubei Key Laboratory of Mine Environmental Pollution Control and Remediation, School of Environmental Science and Engineering, Hubei Polytechnic University, Huangshi 435003, China; 211041@hbpu.edu.cn (H.-L.W.); huangjiexun@hbpu.edu.cn (J.-X.H.); 3 School of Materials Science and Engineering, University of Shanghai for Science and Technology, Shanghai 200093, China; 192432592@st.usst.edu.cn

In the original publication [1], there was a mistake in Figure 6 as published. The authors want to change it to a new one to avoid any possible misleading of the readers. The XRD raw data were not converted into lines in a suitable manner.

The corrected Figure 6 is provided below.

**Figure 6:**
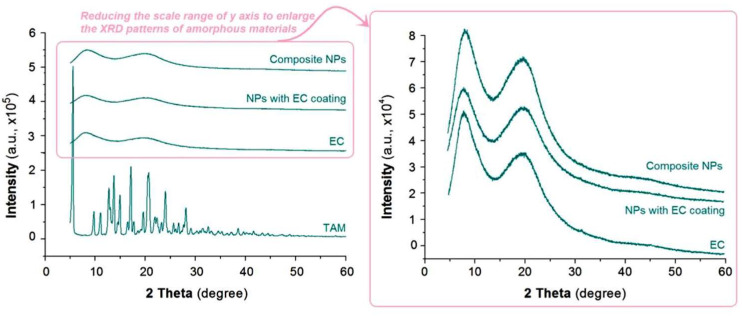
The XRD patterns of the crude materials and also their nanoparticles.

The authors state that the scientific conclusions are unaffected. This correction was approved by the Academic Editor. The original publication has also been updated.

## References

[1] Huang W.-D., Xu X., Wang H.-L., Huang J.-X., Zuo X.-H., Lu X.-J., Liu X.-L., Yu D.-G. (2020). Electrosprayed Ultra-Thin Coating of Ethyl Cellulose on Drug Nanoparticles for Improved Sustained Release. Nanomaterials.

